# Prehypertension and its predictors among older adolescents: A cross-sectional study from eastern Nepal

**DOI:** 10.1371/journal.pgph.0001117

**Published:** 2022-09-28

**Authors:** Jeevan Thapa, Shyam Sundar Budhathoki, Surya Raj Niraula, Sagar Pandey, Nishant Thakur, Paras K. Pokharel

**Affiliations:** 1 Department of Community Health Sciences, Patan Academy of Health Sciences, Lalitpur, Nepal; 2 Faculty of Medicine, Department of Primary Care and Public Health, School of Public Health, Imperial College London, London, United Kingdom; 3 School of Public Health and Community Medicine, B.P. Koirala Institute of Health Sciences, Dharan, Nepal; 4 B.P. Koirala Institute of Health Sciences, Dharan, Nepal; 5 Epidemiology and Disease Control Division, Teku, Kathmandu, Nepal; University of the Witwatersrand, SOUTH AFRICA

## Abstract

Prehypertension is a state of transition between normal blood pressure and hypertension. Adolescent prehypertension is a strong predictor of hypertension in adults and is now considered for cardiovascular intervention or risk reduction. This study was conducted among adolescents to assess the burden of pre-hypertension and its predictors. A cross-sectional study was conducted among grade 11 and 12 students in three districts in eastern Nepal namely Jhapa, Morang and Sunsari. Sampling was done using a multistage stratified proportionate random method. A semi-structured questionnaire adapted from the WHO STEPwise approach to the non-communicable disease risk factor surveillance (STEPS) instrument was used as a study tool after modification and pre-testing in addition to the anthropometric and blood pressure measurements by the investigators. The prevalence of prehypertension was assessed along with the identification of its predictors through multivariable binary logistic regression modelling. A total of 806 participants aged 15 to 19 years, with 57.1% female, participated in the study. Prehypertension was found in 20.8% (24.6% in males and 18.0% in females) of the participants, while 7.1% of them were hypertensive (9.2% males and 5.4% females). Obesity and central obesity were seen among 6.3% and 17.7% of the respondents respectively. Age, sex, ethnicity and obesity were found to be significantly associated with prehypertension. A significant proportion of prehypertension was seen among the adolescent population along with a notable presence of risk factors such as smoking, alcohol consumption, obesity, and eating out. This warrants careful consideration and identification of relevant strategies to reduce the burden of prehypertension via school-based interventions to reduce the modifiable risk factors.

## Introduction

The seventh report of the Joint National Committee on the prevention, detection, evaluation and treatment of high blood pressure (JNC 7) defines normal blood pressure as a systolic blood pressure less than 120 and diastolic blood pressure less than 80mm Hg. Prehypertension, on the other hand, is a state of high normal blood pressure and is defined as a systolic blood pressure of 120–139 mmHg and/or a diastolic blood pressure of 80–89 mmHg in adults aged ≥18 years of age as per JNC 7 [1]. The fourth report on the diagnosis, evaluation and treatment of high blood pressure in children and adolescents by the National High Blood Pressure Education Program Working Group (NHBPEP) defines prehypertension for children and adolescents (˂18 years) as an average systolic blood pressure or diastolic blood pressure levels that are ≥ 90^th^ percentile but <95^th^ percentile for age, gender and height. In addition, BP ≥120/80 mm Hg, even if this figure is <90^th^ percentile, is considered prehypertension in adolescents according to NHBPEP [2].

The World Health Organization (WHO) defines adolescents as those people between 10–19 years of age [3]. In addition to being a transition period to adulthood, adolescence is one of the most dynamic stages of human development where they start to make individual choices and develop behaviours that often persist in adulthood [4]. Nearly two-thirds of premature non-communicable disease (NCD) deaths and one-third of disease burden among adults are associated with conditions or behaviour that began in adolescence including tobacco and alcohol use, physical inactivity and unhealthy diet [5]. Furthermore, there is strong evidence of hypertension in adults has its origin in childhood [6–8]. Adolescent prehypertension is found to be a strong predictor of hypertension in adults. Studies have shown that systolic blood pressure above the age and gender-specific blood pressure values predicted an increased risk of hypertension and metabolic syndrome later in life [9]. Prehypertension, despite being a transition phase between normal blood pressure (BP) and hypertension, has been reported as an independent risk factor for cardiovascular events and stroke [10,11]. The rate of progression of prehypertension to hypertension was found to be 7% per year in a study that examined the longitudinal blood pressure outcomes for adolescents [12].

Prehypertension is reported in a high proportion of adults with a prevalence ranging from 21.9% in China [13] to 52% in Iran [14] in early 2000. These differences in the prevalence of prehypertension could be due to differences in the proportion of risk factors among the population. In China, the mean BMI among male and female participants was 22.55 and 22.99 kg/m^2^ respectively [13]; while in Iran the mean adjusted BMI among male and female participants was 25.2 and 27.4 kg/m^2^ respectively [14]. Among younger population, its prevalence ranges from 9.2–16.4% in South Africa [15,16] to 12.3% - 24.5% in India [17–21]. Early detection of prehypertension has been accepted as a potential opportunity for cardiovascular disease risk reduction. It necessitates lifestyle interventions to prevent or delay the progression to hypertension [1]. In Nepal, the pooled prevalence of pre-hypertension among adults is reported at 35.4% [22]. A single cross-sectional study using the data from two trial cohorts conducted in Sarlahi district of Nepal in the 1990s with participants belonging to the 9–23 year age group, reported the prevalence of prehypertension in the range of 11.6–13.3% [23].

There were recommendations for alignment of hypertension evaluation and treatment across the transition from adolescence to adulthood. American College of Cardiology/American Heart Association (ACC/AHA) and the American Academy of Pediatrics updated and aligned the hypertension definitions in the “2017 High Blood Pressure Clinical Practice Guidelines” [24]. The updated guidelines have eliminated the term “pre-hypertension” and favoured the term “Elevated Blood Pressure” for the blood pressure group between normal and hypertension, and have removed the stage 3 hypertension category. The percentile-based categories for hypertension have been retained at younger ages less than 13 years, while for more than 13 years; the definition has been simplified and is the same as that of adulthood. It defines normal BP as SBP <120 mmHg and DBP <80 mmHg; while elevated blood pressure is defined as SBP 120–129 mmHg and DBP <80 mmHg. Stage 1 Hypertension is defined with SBP 130–139 mmHg or DBP 80-89mmHg; and Stage 2 as SBP ≥ 140mmHg or DBP ≥ 90 mmHg. However, in the Nepali setting, the term pre-hypertension is still being used, as can be seen in many recent studies based on the previous definitions [22,25,26]. We have also adopted the previous definition (based on the JNC 7^th^ and 4^th^ Report) for hypertension categories and assessed the prevalence and risk factors of pre-hypertension. However, there are chances that the burden of pre-hypertension might have been underestimated by the previous definition used in this study.

With increasing evidence that risk factors for prehypertension and hypertension start to develop early in life and literature reporting the increasing prevalence of prehypertension globally, research must be focused on prehypertension among the adolescent population in Nepal. Though there are various studies reporting hypertension and pre-hypertension among adults [22,25,26], there is a dearth of literature reporting on prehypertension among adolescents in Nepal. More seminal research evidence is particularly important as there are initiatives [27,28] aiming to address cardiovascular health among school children in Nepal. More research on adolescents and school children could help build a strong evidence base for justifying interventions and formulation of policies. Hence this study, therefore, aims to assess the burden of prehypertension among adolescents and identify the predictors of prehypertension in Nepal.

## Methods

### Ethics statement

The ethical approval was obtained from the institutional review committee of BP Koirala Institute of Health Sciences (code no. IRC/0876/016). Permission for the study was also sought from the selected schools, along with written consent from participants aged 18 years and above. For younger participants, in addition to written assent from participants, written consent was also obtained from their parents/legal guardians before proceeding with the survey. For parents/legal guardians who were unable to read, information regarding the objectives of the study, expected time duration to complete the survey, an overview of the format of the questionnaire, voluntary participation along with a declaration of confidentiality and anonymity were explained by the investigator beforehand. All of the ethical standards were followed while conducting the study.

### Study design

It was a cross-sectional study done among students attending grades 11 and 12 in eastern terai districts of Nepal, viz Jhapa, Morang and Sunsari. The study was conducted from March 2017 to February 2018.

### Participants

The study participants included adolescent students studying in grades 11 and 12 in public or private institutions who consented to participation. There were a total of 290 eligible public and private institutions running secondary school programs in the Jhapa, Morang and Sunsari districts, with 71,722 students registered for grade 11 and 12 board examinations as per the information provided by Higher Secondary Education Board (HSEB) Office, Biratnagar, Nepal.

### Study size and sampling technique

The sample size was calculated based on a cohort study conducted in Sarlahi, Nepal, where the prevalence of prehypertension was found to be 11.6% among youth including older adolescents [23]. At a 95% confidence interval with a 20% margin of error and inflation of sample size by 10% to address non-response, a final sample size of 806 was reached.

Multistage, stratified proportionate random sampling was done to obtain the representative sample. In the first stage of sampling, eligible public and private institutions in three districts with students studying in grades 11 and 12 were identified. All of those public and private schools were listed separately for each district and assigned unique codes. Subsequently, a serial number was assigned in ascending order of their codes for both public and private schools in each district. This was followed by the second stage of sampling where two computer-generated random numbers representing the serial number of schools were generated for each school type i.e. public and private in each of the three districts. This summed up to a total of 12 schools, four from each district.

**Fig 1:**
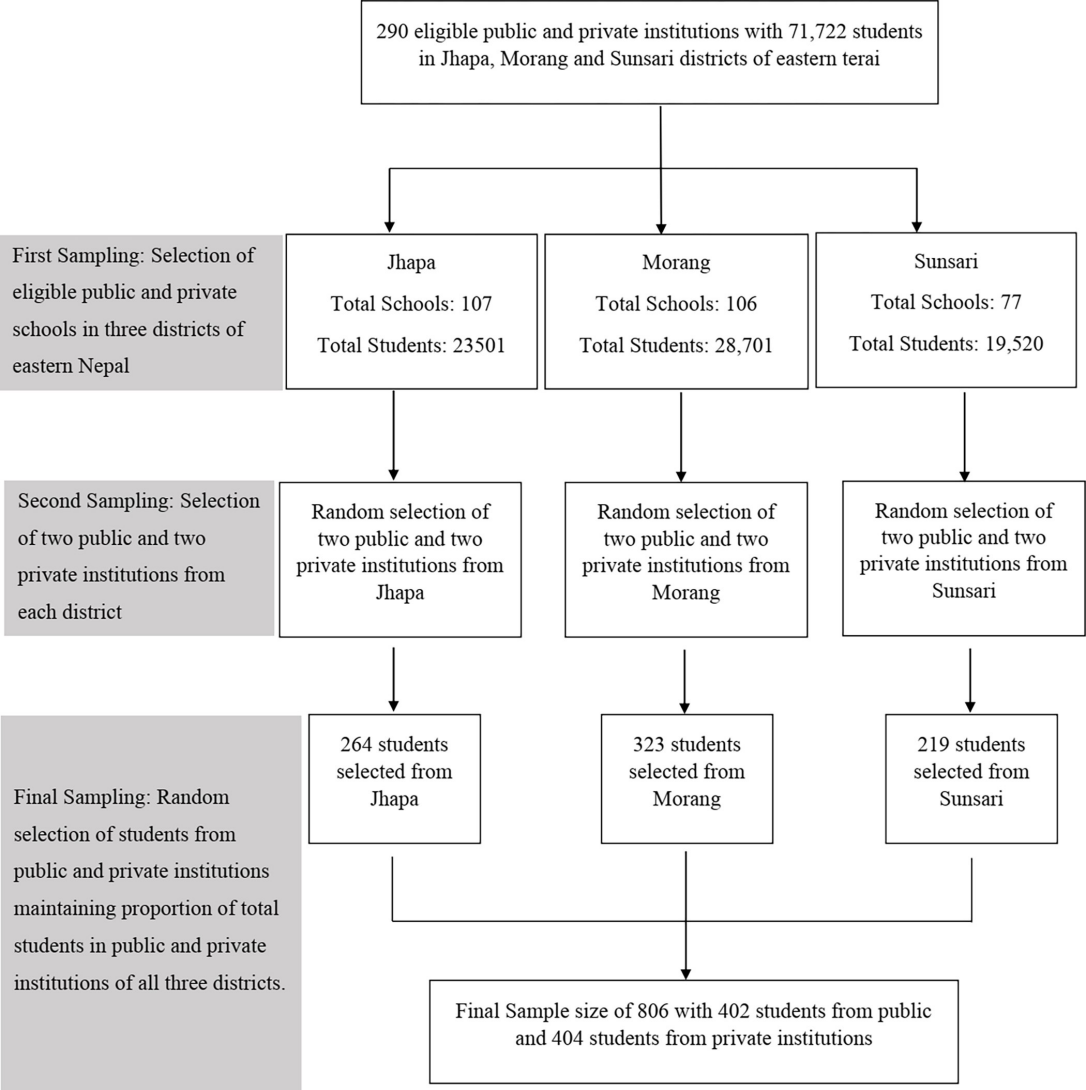
Flowchart depicting the process of multistage stratified proportionate random sampling.

The data obtained from the HSEB, office, Biratnagar regarding the total number of students studying in selected schools was confirmed by contacting the selected schools which were then used to obtain an estimate of the number of participants required from the school. The selected school authority was explained about the study, and permission was obtained to conduct the study. In the final stage of sampling, a list of total students in the selected school was prepared and arranged in ascending order of their roll numbers. Students were listed alphabetically on a first-name basis in case of unavailability of roll numbers. Then a unique serial number was assigned to all students. Finally, computer-generated random numbers, which represented the unique serial number, were used to make a decision on which students to include in the study. A flowchart summarizing the process of sampling is depicted in Fig 1 below.

With the help of the school administration, the selected students were contacted, and explained about the study and a suitable day was picked for data collection, without hampering their study. The institution was visited on the days of data collection and appropriate tools were used for data collection. Those absent on the day of data collection were contacted and scheduled on subsequent days for data collection based on their convenience.

### Study tools

Data collection was done using a pre-tested semi-structured questionnaire along with the measurement of anthropometric parameters and blood pressure of the participants. The semi-structured questionnaire was adapted from the WHO STEPS Instrument for Non-communicable disease risk factor surveillance [29]. Nepali translated version of the questionnaire was used for data collection since students were comfortable answering questions framed in their local language. Native speakers with a good comprehension of the English language initially translated the questionnaire into Nepali which was subsequently translated into English by another person who was not previously acquainted with the English version of the questionnaire. Finally, the original version of the questionnaire in English and the back-translated version into English were matched to ensure the validity of the Nepali translation.

Apart from the socio-demographic characteristics of the participants, the questionnaire collected information regarding maternal education, the use of tobacco and tobacco products, alcohol consumption, dietary habits and salt intake, physical activities and personal as well as family history of high blood pressure and diabetes through face-to-face interviews. Lastly, measurements of height, weight, waist circumference and hip circumference were included under anthropometric measurements. Height was measured with participants in bare feet using the principle of a stadiometer. Weight was measured with a regularly calibrated digital scale on a flat surface and participants were on bare feet without holding onto anything. Waist and hip circumference were measured using the WHO STEPS protocol [30]. A non-stretchable tape was used and the measurements were recorded to the nearest centimetres in all cases. Blood pressure was measured using a calibrated aneroid sphygmomanometer after 15 minutes of rest. Measurement was taken on the right arm in a sitting position following the standard procedure. A total of 3 readings were taken, with a gap of 3 minutes in between each reading, and the mean of the 2^nd^ and 3^rd^ readings was recorded.

The interview as well as anthropometric and blood pressure measurement was done by the investigator himself, using a similar technique in all participants. However, the anthropometric measurement of female participants was done in presence of a female teacher.

### Operational definitions for the variables

Ethnicity: It was categorized into 6 groups as per Health Management Information System (HMIS) classification ethnicity [31].

Type of institution: It was categorized as public if the institution was owned by the government or community, or private if the institution was owned by the private sector as a profit-based institution.

Mother’s literacy: The participant’s mother was considered literate if she was able to read all or part of a sentence.

Smoking and alcohol use: Participant was categorized as ever smoker and ever drank alcohol if they had ever smoked or drank alcohol respectively.

Dietary habit: Adequate serving was considered if their diet considered 5 servings of fruits and vegetables per day. We used a dietary showcard used by WHO STEPS to display the serving sizes of fruits and vegetables to the participants, so they could report their intake.

Type of oil used for cooking: It was categorized as mustard oil, sunflower oil, butter/ghee and others, as reported by the participants.

Adding salt to food: It was defined as the frequency of adding table salt before eating or during eating.

Physical activity: Metabolic equivalent of task (MET score) was calculated for every participant based on the information about the different activities and their duration. Based on MET score, they were labelled as Low level (<600), Moderate level (600 - <3000) and High level (3000 and above).

Eating out: The participants who responded having a meal (breakfast, lunch or dinner) from restaurants/shops on average per week were categorized as eating out, while others were categorized as not to be eating out. Adolescents tend to eat fast food such as chowmein (fried noodles), momo (dumplings), burgers, pizza, etc. along with soft drinks [32] as they eat out, and the culture of eating out has been increasing among adolescents in our country.

Family history of hypertension or diabetes: It was defined by the presence of hypertension or diabetes in parents, grandparents or siblings in their family.

Raised blood glucose: The participants were said to have raised blood glucose if they ever had their blood glucose measured, and were told by health care workers that they had raised blood glucose.

Prehypertension and hypertension: It was defined based on the Fourth Report on the Diagnosis, Evaluation and T/t of High blood pressure in children and adolescents by NHBPEP for those aged less than 18 years [2]. For those who were aged 18 years and older, JNC 7 criteria were used [1] (Table 1).

**Table 1:** Blood pressure classification based on the 4th report and JNC 7.

Blood Pressure Classification	For <18 years	For 18 years and older	
	SBP or DBP	SBP (mm Hg)	DBP (mm Hg)
**Normal**	< 90th percentile	<120	And <80
**Prehypertension**	≥ 90th and < 95th percentile	120–139	Or 80–89
**Hypertension**	≥ 95th percentile	≥ 140–159	Or ≥ 90

Body Mass Index (BMI): BMI was calculated, and interpreted according to BMI for age growth charts for boys and girls aged 2 to 20 years as per the CDC paediatrics growth charts [33]. BMI-for-age at or above the 95th percentile was categorized as obese and that between 85th and 95th percentile as overweight.

Central obesity: A waist-height ratio of ≥ 0.5 was used to define central obesity for both males and females.

### Statistical analysis

Data were entered in Microsoft excel, cleaned and coded. It was then exported into Statistical Package for Social Sciences (SPSS) version 16 for analysis. Categorical data were presented in counts and percentages, while continuous data were presented in mean, standard deviation, median and quartiles as appropriate.

Prevalence of prehypertension and hypertension was calculated, and the association of prehypertension with predictors was assessed excluding the hypertensive population. A Chi-square test was applied to assess the association of categorical predictor variables such as age group, sex, ethnicity, etc. with prehypertension. An independent sample t-test was used to compare continuous variables such as age (in years), BMI, sleep duration, etc among the normal and pre-hypertensive groups (if they differed statistically significantly). If these continuous variables were not normally distributed, the Mann-Whitney U test was used to compare the differences between these variables among the normal and hypertensive groups. Karl Pearson’s correlation coefficient was obtained to assess the correlation between age, BMI, waist-height ratio, waist-hip ratio, systolic blood pressure, diastolic blood pressure and mean arterial pressure.

Univariable binary logistic regression analysis of predictors was done to calculate the relation of predictors with prehypertension. Crude odd’s ratio and its 95% confidence interval were calculated along with its p-value. All the variables with corresponding p-value <0.25 were tested for collinearity to consider for the first multivariable model. All the non-collinear variables (with Variation Inflation Factor <2) were then taken in the first multivariable binary logistic regression model. All the ten variables (viz. age, sex, ethnicity, mother’s literacy, smoking, eating outside the home, sleep duration, family h/o HTN, obesity and central obesity) were found to be non-collinear and were considered in the first model. Deviance of the first model (i.e. -2 log-likelihood) was calculated. The non-significant predictors from the model were removed stepwise, by comparing the changes in the -2 log-likelihood of the model. If the removal of a variable brought significant change in the model, then the variable was retained, or else dropped from the model. The family history of hypertension was purposefully retained in the model due to its clinical significance despite no statistical significance in the model based on our data. The final model (model II) thus obtained using multivariable binary regression analysis consisted of six predictors (age, sex, ethnicity, eating outside the home, family history of hypertension and obesity) of prehypertension.

## Results

The study included 806 participants, who had been studying in public and private institutions in grades 11 and 12 in the three terai districts of the eastern region. All the students selected for the study consented to study and participated. Data was collected from all these students (in first or subsequent visits) with no non-response.

The age of the participants ranged from 15 to 19 years, with a mean (± SD) age of 17.3 (± 0.9) years. There were 57.1% female participants, while 51.0% were Janjati by ethnicity. More than half (52.1%) of students were from public institutions. Among the participants, 22% were ever smokers, while 37.3% were ever drinkers of alcohol. Only 3.2% of them had adequate servings of fruits and vegetables. Obesity based on BMI was seen among 6.3% of them, while 17.7% were found to be centrally obese (Table 2).

**Table 2:** Distribution of sociodemographic factors and risk factors among the participants (n = 806).

Characteristics		Frequency	Percentage
**Age**	**<18 years**	489	60.7
	**≥18 years**	317	39.3
	**Mean ± SD (years)**	17.3 ± 0.9	
**Sex**	**Female**	460	57.1
	**Male**	346	42.9
**Ethnicity**	**Janjati**	411	51.0
	**Others**	395	49.0
**Type of institution**	**Public**	420	52.1
	**Private**	386	47.9
**Mothers education**	**Literate**	539	66.9
	**Illiterate**	267	33.1
**Ever smoker**	**No**	629	78.0
	**Yes**	177	22.0
**Ever drank alcohol**	**No**	505	62.7
	**Yes**	301	37.3
**Adequate servings**	**No**	780	96.8
	**Yes**	26	3.2
**Type of oil used in cooking**	Mustard oil	355	44.0
	Sunflower oil	420	52.1
	Butter‎/Ghee	8	1.0
	Others	23	2.9
**Adding salt to food**	Always‎/Often	76	9.4
	Sometimes‎/Rarely‎/ Never	730	90.6
**Physical activity (MET score)**	Low level (<600)	330	40.9
	Moderate level (600 - <3000)	384	47.6
	High level (3000 and above)	92	11.4
**Eating outside home**	**No**	198	24.6
	**Yes**	608	75.4
**Sleep duration**	**≥ 7 hours**	602	74.7
	**< 7 hours**	204	25.3
	**Mean ± SD (hours)**	7.37 ± 1.29	
**Family history of HTN**	**No**	555	68.9
	**Yes**	251	31.1
**Family history of DM**	**No**	715	88.7
	**Yes**	91	11.3
**Raised blood glucose (n = 86)^#^**	No	33	86.8
	Yes	5	13.2
**Obesity**	**No**	755	93.7
	**Yes**	51	6.3
**Central obesity**	**No**	663	82.3
	**Yes**	143	17.7
**Total**		806	100.0

^#^ Only 86 participants ever had their blood glucose checked.

**Fig 2:**
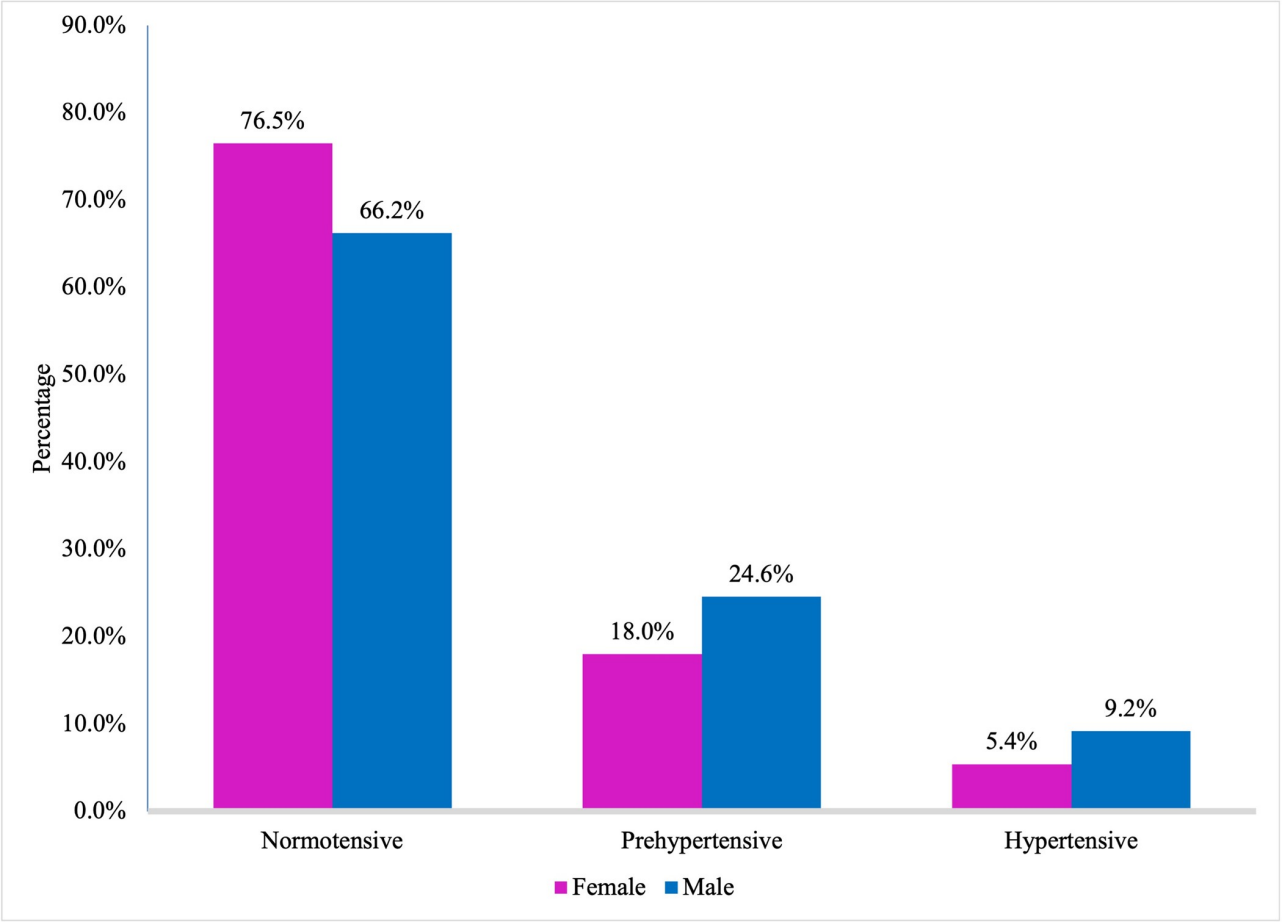
Prevalence of prehypertension and hypertension stratified by sex (n = 806).

Prehypertension was found to be present among 20.8% (168/806) of them with 24.6% in males and 18.0% in females, while hypertension was observed among 7.1% (57/806) of them with 9.2% in males and 5.4% in females (Fig 2).

In the bivariate analysis of factors associated with prehypertension, age, sex, ethnicity, smoking, eating outside the home, hypertension in the family, obesity and central obesity were found to be statistically significant (p < 0.05). There were significant differences in means of age, BMI, waist-hip ratio and waist-height ratio among the pre-hypertensive and normal populations. (Table 3).

**Table 3:** Socio-demographic characteristics and biophysical measurements of participants by pre-hypertension status (n = 749).

Characteristics		Prehypertension		Total	p-value
		Yes (n = 168)	No (n = 581)		
**Age**	**<18 years**	74(16.2%)	384(83.8%)	458(100%)	<0.001
	**≥18 years**	94(32.3%)	197(67.7%)	291(100%)	
	**Mean ± SD**	17.6 ± 0.9	17.23 ± 0.9	17.32± 1.0	
**Sex**	**Female**	83(19.1%)	352(80.9%)	435(100%)	0.010
	**Male**	85(27.1%)	229(72.9%)	314(100%)	
**Ethnicity**	**Janjati**	107(28.1%)	274(71.9%)	381(100%)	<0.001
	**Others**	61(16.6%)	307(83.4%)	368(100%)	
**Type of institution**	**Public**	83(21.1%)	311(78.9%)	394(100%)	0.346
	**Private**	85(23.9%)	270(76.1%)	355(100%)	
**Mother’s education**	**Literate**	105(20.9%)	398(79.1%)	503(100%)	0.145
	**Illiterate**	63(25.6%)	183(74.4%)	246(100%)	
**Ever smoker**	**No**	118(20.2%)	465(79.8%)	583(100%)	0.007
	**Yes**	50(30.1%)	116(69.9%)	166(100%)	
**Ever drank alcohol**	**No**	102(21.6%)	370(78.4%)	472(100%)	0.483
	**Yes**	66(23.8%)	211(76.2%)	277(100%)	
**Adequate servings**	**No**	164(22.7%)	560(77.3%)	724(100%)	0.433
	**Yes**	4(16.0%)	21(84.0%)	25(100%)	
**Total servings (per day)**	**Median (Q1, Q3)**	1.57 (0.86, 2.55)	1.43 (0.96, 2.43)	1.43 (0.86, 2.43)	0.739^#^
**Adding salt to food**	Always‎/ Often	18 (25.7%)	52 (74.3%)	70 (100%)	0.489
	Sometimes‎/ Rarely‎/ Never	150 (22.1%)	529 (77.9%)	679 (100%)	
**Type of oil used in cooking**	Mustard oil	73 (22.1%)	258 (77.9%)	331 (100%)	0.884*
	Sunflower oil	88 (22.7%)	299 (77.3%)	387 (100%)	
	Butter‎/Ghee	1 (12.5%)	7 (87.5%)	8 (100%)	
	Others	6 (26.1%)	17 (73.9%)	23 (100%)	
**Physical activity (MET score)**	Low level (<600 MET)	66 (21.4%)	243 (78.6%)	309 (100%)	0.732
	Moderate level (600 - <3000)	80 (22.7%)	273 (77.3%)	353 (100%)	
	High level (3000 and above)	22 (25.3%)	65 (74.7%)	87 (100%)	
**Eating outside home**	**No**	30(16.4%)	153(83.6%)	183(100%)	0.024
	**Yes**	138(24.4%)	428(75.6%)	566(100%)	
**Sleep duration (hrs)**	**≥ 7 hours**	117(20.8%)	445(79.2%)	562(100%)	0.067
	**<7 hours**	51(27.3%)	136(72.7%)	187(100%)	
	**Mean ± SD**	7.21 ± 1.14	7.42 ± 1.31	7.37 ± 1.28	
**HTN in family**	**No**	104(20.1%)	413(79.9%)	517(100%)	0.023
	**Yes**	64(27.6%)	168(72.4%)	232(100%)	
**Diabetes in family**	**No**	146(21.9%)	519(78.1%)	665(100%)	0.381
	**Yes**	22(26.2%)	62(73.8%)	84(100%)	
**Raised blood glucose (n = 86)**	No	6 (20%)	24 (80%)	30 (100%)	0.395*
	Yes	1 (50%)	1 (50%)	2 (100%)	
**Waist hip ratio**	**Mean ± SD**	0.81 ± 0.07	0.79 ± 0.06	0.8 ± 0.06	0.015^##^
**Waist height ratio**	**Mean ± SD**	0.45 ± 0.05	0.43 ± 0.04	0.43 ± 0.05	<0.001^##^
**BMI (kg/m2)**	**Mean ± SD**	21.41 ± 3.25	19.55 ± 2.95	19.97 ± 3.12	<0.001^##^
**Obesity**	**No**	149(21.2%)	555(78.8%)	704(100%)	0.001
	**Yes**	19(42.2%)	26(57.8%)	45(100%)	
**Central obesity**	**No**	145(21.2%)	540 (78.8%)	685(100%)	0.007
	**Yes**	23(35.9%)	41(64.1%)	64(100%)	
**Total**		168(22.4%)	581(77.6%)	749(100%)	

*Fisher Exact test used.

^#^ Mann-Whitney U test applied.

^##^Independent t-test applied.

Age showed a weak positive correlation with systolic blood pressure and mean arterial pressure, which was statistically significant. BMI and waist height ratio also showed weak positive correlation with systolic blood pressure, diastolic blood pressure and mean arterial pressure, and the correlation was statistically significant (p < 0.01) (Table 4).

**Table 4:** Correlation of anthropometric measures with blood pressure (n = 749).

Characters	BMI	Waist hip ratio	Waist height ratio	SBP	DBP	MAP
**Age**	.125**	0.069	0.054	.115**	0.035	.076*
**BMI**	1	.204**	.599**	.224**	.188**	.227**
**Waist hip ratio**	.204**	1	.623**	.123**	0.028	.075*
**Waist height ratio**	.599**	.623**	1	.158**	.090*	.132**
**SBP**	.224**	.123**	.158**	1	.580**	.843**
**DBP**	.188**	0.028	.090*	.580**	1	.927**
**MAP**	.227**	.075*	.132**	.843**	.927**	1

*p < 0.05.

**p < 0.01.

**Fig 3:**
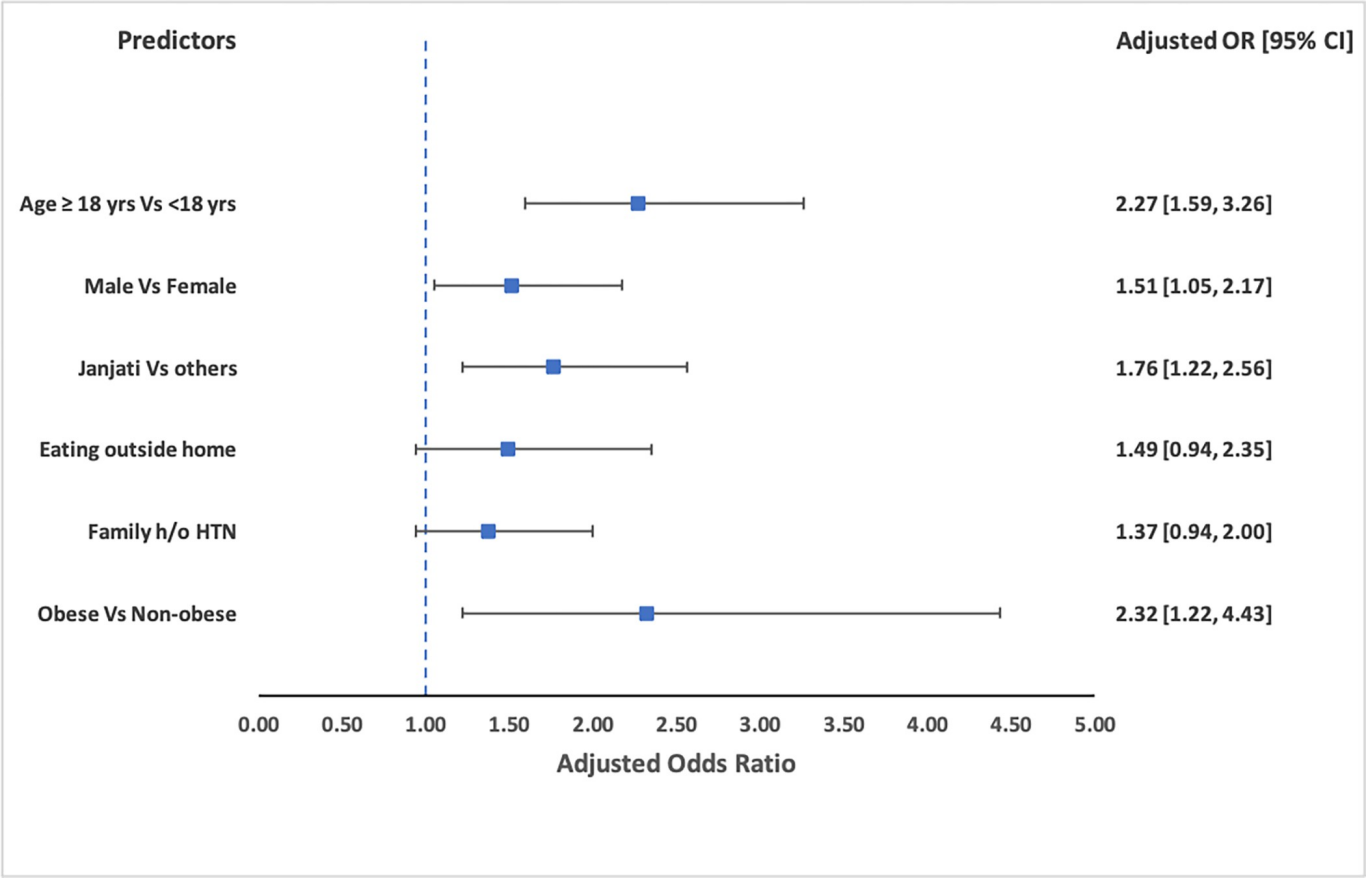
Adjusted odds ratio of predictors of PHTN based on final model (Model II).

In the final multivariable model (model II), participants aged ≥ 18 years were found to be 2.27 (95% CI 1.59, 3.26) times likely to have hypertension than those aged less than 18 years (p <0.001). Males were found to be 1.51 times more likely to have prehypertension than females. Participants belonging to the Janjati ethnicity were 1.76 times more likely to be pre-hypertensive than others. Pre-hypertensive was also significantly higher among those who ever ate outside the home, had family history of hypertension, or were obese. (Table 5) The adjusted odd’s ratio obtained for predictors in the final model has been shown in Fig 3.

**Table 5:** Univariable and multivariable regression analysis of predictors of prehypertension (n = 749).

Predictors	Univariable regression		Model I		Model II	
	OR (95% CI)	p-value	AOR (95% CI)	p-value	AOR (95% CI)	p-value
**Age ≥ 18 years**	2.48 (1.75, 3.51)	<0.001	2.21 (1.54, 3.17)	<0.001	2.27 (1.59, 3.26)	<0.001
**Male sex**	1.57 (1.11, 2.22)	0.010	1.41 (0.93, 2.15)	0.107	1.51 (1.05, 2.17)	0.027
**Janjati ethnicity**	1.97 (1.38, 2.8)	<0.001	1.7 (1.16, 2.49)	0.006	1.76 (1.22, 2.56)	0.003
**Illiterate mother**	1.30 (0.91, 1.87)	0.145	1.25 (0.86, 1.83)	0.247	-	-
**Ever smoker**	1.70 (1.15, 2.5)	0.007	1.17 (0.73, 1.87)	0.504	-	-
**Eating outside home**	1.64 (1.06, 2.54)	0.025	1.49 (0.94, 2.35)	0.091	1.49 (0.94, 2.35)	0.087
**Sleep less than 7 hours**	1.43 (0.97, 2.09)	0.068	1.22 (0.81, 1.82)	0.337	-	-
**Family h/o HTN**	1.51 (1.06, 2.17)	0.024	1.37 (0.94, 2.01)	0.105	1.37 (0.94, 2)	0.103
**Obesity**	2.72 (1.47, 5.05)	0.002	1.75 (0.83, 3.7)	0.140	2.32 (1.22, 4.43)	0.011
**Central obesity**	2.09 (1.21, 3.59)	0.008	1.66 (0.86, 3.23)	0.134	-	-
**Model summary**	**Constant**		0.066	<0.001	0.075	<0.001
	**- 2log likelihood**		736.361		741.052	
	**Cox & Snell R Square**		0.078		0.072	
	**Nagelkerke R Square**		0.119		0.111	

## Discussion

With the rising incidence of NCD and ongoing change in epidemiological determinants that favour NCD, the need to assess the burden of NCD and its predictors is ever increasing, especially among the adolescent age group. This study accordingly reported the burden of prehypertension and its predictors among students of grades 11 and 12 in Jhapa, Morang and Sunsari districts of Nepal. One-fifth of the adolescents (20.8%) in the study were found to be pre-hypertensive, while 7.1% of them were found to be hypertensive. About 6.3% of them were found to be overweight and obese whereas 17.7% had central obesity. A quarter of the study participants were found to be ever smokers and a third of them ever drank alcohol. We found a significant association between age, sex, ethnicity and obesity with prehypertension.

The prevalence of pre-hypertension in this study was found to be similar to the study done among 250 medical students of KIST medical college in Nepal, where the prevalence of pre-hypertension was also 20.8%. However, for the study, the average of two readings was recorded and classified, and the study participants’ age ranged from 17 years to 25 years [25]. The prevalence of prehypertension in this study also correlated with the prevalence reported in a school-based study in Kerala and Shimla, India where the respective prevalence was 24.5% and 22.3% [20,21]. On the other hand, the reported prevalence of prehypertension and hypertension during the follow-up study of Nepal Nutrition Intervention Project Sarlahi (NNIPS) cohorts aged 9–23 years was comparatively lower, with prehypertension ranging from 11.6% to 13.3% and hypertension ranging from 4.7% to 6.4% [23]. An explanation for this discrepancy might be due to the increasing trend of prehypertension and hypertension with an increase in risk factors prevalence in the current scenario compared to that from 2006 to 2008 when the study was conducted.

The study has reported an increase in the proportion of prehypertension in a higher age group, with age being positively and significantly correlated with systolic blood pressure and mean arterial pressure. Age is statistically significant in the final model in our study. This was, however, an expected finding since an age-related increase in blood pressure has been a proven concept in human ageing [34,35]. A higher proportion of prehypertension among male adolescents obtained from the final model in this study corroborates with a study in Kerala with a similar risk reported among the adolescents [21]. The higher blood pressure in males than females is attributed to differences in cardiovascular effects of testicular and ovarian hormones as well as the role of sex chromosomes [36].

The proportion of prehypertension was found to be highest among Janjati ethnic group in this study. Although studies reporting a similar prevalence of prehypertension among Janjati in different study settings are lacking, several studies have reported similar findings among ethnic groups having a closer resemblance to Janjati ethnic groups of eastern Terai. For example, the Dhulikhel heart study conducted between 2013 to 2015 among 752 participants reported a higher prevalence of prehypertension among Newars (54.8%) compared to Brahmin/Chhetri (39.7%) and others (48.5%) [26]. Similarly, a community-based cross-sectional study of hypertension in Duwakot, Bhaktapur by Vaidya A. reported that Tibeto-Burmans (which include Tamang, Rai, Limbu, Sherpa, etc.) had a higher prevalence of hypertension (25.3%) compared to Indo-Aryans (which includes Brahmin, Chhetri, people of terai and Tharu) where the number was only 14%. An independent ethnic variation in the blood pressure distribution among the Nepalese population possibly acting independently of various lifestyle determinants of hypertension was hypothesized as a reason for the findings of the study [37]. In addition, various socio-cultural practices in different ethnicities, like the obligatory provision of alcohol during festivals, might have a role to play in the observed findings.

This study reported a significant positive correlation between BMI and systolic blood pressure and diastolic blood pressure. The proportion of prehypertensive overweight/obese adolescents in this study was double as compared to normal or underweight adolescents. In addition, overweight/obese individuals had an adjusted odds of 2.32 (p-value- 0.011) than those with normal or underweight. The effect of an increase in blood pressure with an increase in BMI has been extensively reported in the medical literature [38–40]. Furthermore, a significant positive correlation of BMI with systolic blood pressure and diastolic blood pressure among children and adolescents is also reported in studies conducted in various states of India [17,21,41–43]. In addition to the association of BMI with high blood pressure, it was also reported to be strongly associated with the development of atherosclerotic lesions in the aorta and coronary arteries [44]. This signifies the importance of early detection and management of prehypertension and its risk factors to prevent or delay the development of atherosclerotic changes. Lastly, waist-hip ratio and waist-height ratio also showed a significant positive correlation between systolic blood pressure and diastolic blood pressure. The positive association between waist-height ratio and the presence of hypertension was also demonstrated in a cross-sectional study among adolescents aged 10–17 years in Brazil [45]. In addition, in a meta-analysis of 24 cross-sectional studies and ten prospective studies with more than 500,000 participants, the waist-height ratio was found to be more favourable than BMI to detect cardiometabolic risks, elevated blood pressure being one of them [46]. This helps further emphasize the seriousness of the positive association of waist-height ratio with systolic blood pressure and diastolic blood pressure in this study and the need for immediate and long term intervention programs to address the issue.

### Limitations

Blood pressure measurement tends to decrease with repeated measures over a single visit, as well as repeated measures in different visits. So, it is suggested to obtain multiple measurements over time before diagnosing hypertension [47]. However, in this study, all three measurements were taken in a single visit. This might have overestimated the burden of pre-hypertension.

Due to the cross-sectional design of the study, factors associated with prehypertension in the study need to be carefully interpreted. Nepal is a geographically and demographically diverse country, the finds of this study which is primarily conducted among adolescents in the eastern districts of the country might affect the generalizability of the findings.

## Conclusion

Prehypertension was notably present alongside hypertension. Modifiable risk factors such as smoking, eating outside the home, BMI and central obesity found in this study urges careful attention to these factors in health promotion activities focusing on the adolescents in Nepal. Alongside considering packages for managing prehypertension and prehypertension targeting adolescents, future research and health policies need to consider adolescents as an important population for hypertension control programs.

## Supporting information

S1 Text Adapted WHO STEPS questionnaire (English version). (DOCX)
