# Supplementary material for: Prehypertension and its predictors among older adolescents: A cross-sectional study from eastern Nepal
Source: PLOS Glob Public Health. 2022 Sep 28;2(9):e0001117. doi: 10.1371/journal.pgph.0001117 (PMC10021258; doi:10.1371/journal.pgph.0001117)
Supplement: S1 Text — (DOCX) [file pgph.0001117.s001.docx]

Prehypertension and its predictors among adolescents: A cross-sectional study from eastern Nepal

**Participant ID:**

**PART 1: Identifying Information**

1) Name of the respondent ………………………… (Not to be included for analysis)

2) Age 3) Sex 4) Class: 1. XI 2. XII

5) Stream: 1. Science 2. Humanities 3. Management 4. Others (specify) …

6) Ethnicity: 7) Religion:

8) Mobile No - (not to be included for analysis)

9) Residential status- 1) Permanent (own home) 2) Rented

10) Maternal education:

**This page is stored securely and separately from part 2**

**Participant ID: (Same as above)**

**PART 2:**

**Behavioral Measurements**

**A. Tobacco use**

1. Do you currently smoke any tobacco products, such as cigarettes, cigars, pipes, bidis, hukkahs or tamakhus?

1. Yes 2. No (If no Q 7)

2. Do you currently smoke tobacco products daily?

1. Yes 2. No

3. How old were you when you first started smoking?

Age (years) Don’t know 77

4. On average, how many of the following products do you smoke each day/week?

1. Manufactured cigarettes 2. Hand-rolled cigarettes 3. Pipes full of tobacco

4. Cigars, cheroots, cigarillos **5.** Other (please specify):

(If less than daily, record weekly)

5. During the past 12 months, have you tried to stop smoking? 1. Yes 2. No

6. During any visit to a doctor or other health worker in the past 12 months, were you advised to quit smoking tobacco? 1. Yes 2. No 3. No visit

7. In the past, did you **ever smoke** any tobacco products? 1. Yes 2. No

8. In the past, did you **ever smoke daily**? 1. Yes 2. No

9. How **long ago** did you stop smoking? ………..

10. Do you **currently use any smoke-less tobacco products**? 1. Yes 2. No

11 Do you **currently use smokeless tobacco products daily**? 1. Yes 2. No

12. In the **past**, did you **ever use** smokeless tobacco products daily? 1. Yes 2. No

**B. Alcohol consumption:**

1. Have you **ever** consumed an alcoholic drink such as beer, wine, Spirits, jaad , rakhhshi, tongba? 1. Yes 2. NO

2. Have you consumed an alcoholic drink within the **past 12 months**? 1. Yes 2. No

3. During the past 12 months, **how frequently** have you had at least one alcoholic drink?

1. Daily 2. 5-6 days/wk 3.1-4 days/wk 4.1-3 days /month

5. Less than once a month

5. Have you consumed an alcoholic drink within the **past 30 days**? 1. Yes 2. No

6. During the past 30 days, on how many **occasions** did you have at least one alcoholic drink? Number 77. Don’t know

7. During the past 30 days, when you drank alcohol, **on average** how many **standard alcoholic drinks** did you have during one drinking occasion? Number Don’t know 77

8. During the past 30 days, what was the **largest number** of standard alcoholic drinks did you have during one drinking occasion? And how many such occasions?

9. Past 30 days how often you consumed with meals?

1. Usually with meals 2. Sometimes 3. Rarely 4. Never

**C. Diet**

1. In a typical week, on how may days do you eat fruit? No of days Don’t know 77

2. Number of serving on any of those days? No of servings Don’t know 77

3. In a **typical week**, on how many days do you **eat vegetables**?

No of days……….. Don’t know 77

4. How many servings of vegetables do you eat on one of those days?

No of servings…… Don’t know 77

5. Type of oil used for cooking?

1. Mustard oil 2. Refined vegetable oil 3. Sunflower oil 4. Butter or ghee

5. Others (specify)

6. On average, how many meals per week do you eat that were not prepared at home?

(By meal, I mean breakfast, lunch and dinner) Number of meals ….

**D. Salt**

1. How often do you **add salt** to your food before you eat it or as you are eating it?

1. Always 2. Often 3. Sometimes 4. Rarely 5. Never 77. Don’t know

2. How often do you eat processed food high in salt?

1. Always 2. Often 3. Sometimes 4. Rarely 5. Never 77. Don’t know

3. How much salt do you think you consume?

1. Far too much 2. Too much 3. Just the right amount 4. Too little 5. Far too little 77. Don’t know

4. Do you think too much salt in your diet could cause a serious health problem?

1. yes 2. No 77. Don’t know

5. How important to you is **lowering salt** in your diet?

1. Very important 2. Somewhat important 3. Not at all important 77. Don’t know

6. Which type of salt do you use?

1. Crystal salt 2.Powdered salt without logo 3. Ayo Noon 4. Others ( Specify)

**E. Physical Activity**

1. How much time do you spend doing moderate intensity activities (housework, domestic chores, kitchen work, gardening) on a typical day?

2. Do you walk or use a bicycle (pedal cycle) for at least 10 minutes continuously to get to and from places? 1. Yes 2. No

3. How many days in a typical week? No of days

4. In a typical week, on how many days do you walk on bicycle for at least 10 minutes continuously?

5. Do you do any vigorous-intensity sports, fitness or recreational (leisure) activities that cause large increases in breathing or heart rate like [running or football] for at least 10 minutes continuously? 1. Yes 2. No

6. In a typical week, on how many days do you do vigorous-intensity sports, fitness or recreational (leisure) activities? Number of days

7. How much time do you spend doing vigorous-intensity sports, fitness or recreational activities on a typical day?

………

8. Do you do any moderate-intensity sports, fitness or recreational (leisure) activities that cause a small increase in breathing or heart rate such as brisk walking, [cycling, swimming, volleyball] for at least 10 minutes continuously?

1. Yes 2. No

9. In a typical week, on how many days do you do moderate-intensity sports, fitness or recreational (leisure) activities?

Number of days

10. How much time do you spend doing moderate-intensity sports, fitness or recreational (leisure) activities on a typical day?

………….. (hours/minutes)

11. How much time in a day, do you usually spend watching tv or using internet/facebook/chatting playing video games/games in mobile? .........

**F. History of raised blood pressure**

1. Have you ever had your blood pressure measured by a Health worker? 1. Yes 2. No

2. Have you been told that you have raised blood pressure? 1. Yes 2. No

3. Are you currently taking any medication for your HTN? 1. Yes 2. No

4. Is there raised blood pressure in your parents/grandparents/siblings? 1. Yes… (specify.. whom) 2. No

5. Are they taking medication for raised blood pressure? 1. Yes 2. No

**G. History of Diabetes**

1. Have you ever had your blood glucose measured by a doctor or other health worker?

1. Yes 2. No

2. Have you ever been told that you have raised blood glucose? 1. Yes 2. No

3. Are you taking any medication for diabetes? 1. Yes 2. No

4. Are your parents/grandparents/siblings suffering from diabetes?

1. Yes… (specify. whom) 2. No

5. Are they taking medication for diabetes? 1. Yes 2. No

**H. Physical measurement:**

1. Height

2. Weight

3. Waist Circumference

4. Hip Circumference

5. Blood Pressure: Reading 1. Reading 2. Reading 3.
